# Supplementary material for: Identification and functional analysis of non-coding regulatory small RNA FenSr3 in Bacillus amyloliquefaciens LPB-18
Source: PeerJ. 2023 May 15;11:e15236. doi: 10.7717/peerj.15236 (PMC10194069; doi:10.7717/peerj.15236)
Supplement: Supplemental Information 4 [file peerj-11-15236-s004.zip › KO/CK-vs-T1_map/map04112.html]

KEGG PATHWAY: Cell cycle - Caulobacter - Reference pathway


|  |  |
| --- | --- |
| **Cell cycle - Caulobacter - Reference pathway** |  |

[
Pathway menu
| Organism menu
| Pathway entry
| Show description
| User data mapping
]

Cell cycle progression of Caulobacter crescentus is implemented by an elaborate transcriptional regulatory network. During the life cycle, Caulobacter divides asymmetrically producing two distinct cell types with specialized development programs. The sessile stalked cell immediately initiates a new round of division cycle, whereas the motile swarmer cell remains in the G1 phase for a defined interval before differentiating into the stalked cell and entering into the S phase. The division process is centered around three master regulator proteins, DnaA, GcrA, and CtrA. DnaA and GcrA together drive the synthesis of proteins needed to complete DNA replication. CtrA controls the transcription of a diverse group of genes, which are required for essential morphological, metabolic, and cell cycle events.

|  |  |  |
| --- | --- | --- |
| Reference pathway | 184% 150% 122% 100% 82% 67% 55% | 图片下载 |
